# HIV-1 interacts with human testicular germ cells *in vitro*

**DOI:** 10.1186/1742-4690-10-S1-P26

**Published:** 2013-09-19

**Authors:** Dominique Mahé, Claire Deleage, Giulia Matusali, Anne-Pascale Satie, Karim Bensalah, Laurence Guézenec, Bernard Jégou, Nathalie Dejucq-Rainsford

**Affiliations:** 1 INSERM U1085-IRSET, Rennes, France; 2 Université de Rennes I, Rennes, France; 3 SFR Biosit, Rennes, France; 4 Centre Hospitalier Universitaire de Pontchaillou, Service Urologie, Rennes, France; 5 Centre Hospitalier Universitaire de Pontchaillou, Centre de coordination des prélèvements, Rennes, France

## Background

The recent reports of the endogenisation of SIV in primates demonstrate that lentiviruses can infect the germinal lineage. Testicular germ cells (TGC) of both infected men and macaques have been shown to harbor HIV-1/SIV nucleic acids and/or proteins *in situ* by several teams including ours. Although HIV-1 binds but cannot enter isolated human spermatozoa, viral DNA has been detected in a few sperm cells from infected men, suggesting a clonal infection of their progenitors, the TGC. In this context, our investigation focused on the ability of human TGC to interact with HIV-1.

## Materials and methods

TGC were isolated from normal human testes obtained at autopsy or following orchidectomy by enzymatic and mechanical dissociations. The purity of the preparation, as well as the expression of HIV receptors, was evaluated in flow cytometry. HIV-1 (R5 SF162 and primary strains, X4 IIIB and primary strains) binding on TGC untreated or treated with pronase (to remove proteins from the surface) was evaluated by p24 ELISA. The involvement of cellular HIV receptors and of the viral envelope protein Gp120 (env) in HIV-1 attachment was assessed.

## Results

TGC preparations, composed of haploid spermatids, tetraploid spermatocytes and diploid spermatogonia and spermatocytes, were on average 94% pure and contained less than 4% of contaminating testicular somatic cells and 2% of CD45+ leukocytes.

As expected, TGC were devoid of CD4. However, they expressed at their membrane heparan sulfate proteoglycans, the mannose receptor and galactocerebroside, as well as CCR3.

Both HIV-1 R5 and X4 strains bound TGC in a dose dependent manner, at either 4° or 37°C. Protease treatment of the cells prior and post HIV exposure drastically reduced viral binding. Heparan sulfate proteoglycans played a predominant role in HIV-1 attachment, as shown by the inhibitory effect of either heparin competitor or heparinase treatment of the cells. The mannose receptor also contributed to a lesser extent to HIV-1 binding with TGC. Gp120 neutralizing antibodies reduced HIV-1 attachment to germ cells. Similarly, the binding of *env*-depleted HIV-1 pseudovirus to germ cells was decreased when compared with *env*-positive pseudovirus.

## Conclusions

Isolated human testicular germ cells express several alternative HIV receptors and support the attachment of HIV-1 R5 and X4 strains. Heparan sulfate proteoglycans and mannose receptor are mainly involved in the capture of HIV-1 by germ cells. HIV-1 binding to germ cells is partly mediated by the env protein. Whether testicular germ cells can support viral entry and further steps of the viral cycle (e.g. reverse transcription, integration) is under investigation.

